# Utilisation of healthcare in children born to lymphoma survivors in Sweden

**DOI:** 10.2340/1651-226X.2025.43950

**Published:** 2025-11-23

**Authors:** Joshua P. Entrop, Viktor Wintzell, Caroline E. Dietrich, Ingrid Glimelius, Tarec C. El-Galaly, Karin E. Smedby, Sandra Eloranta

**Affiliations:** 1 Clinical Epidemiology Division, Department of Medicine Solna, Karolinska Institutet, Stockholm, Sweden; 2 Department of Immunology, Genetics and Pathology, Cancer Precision Medicine, Uppsala University, Uppsala, Sweden; 3 Department of Clinical Medicine, Aarhus University, Aarhus, Denmark; 4 Department of Molecular Medicine, Aarhus University Hospital, Aarhus, Denmark; 5 Department of Clinical Epidemiology, Aarhus University Hospital, Aarhus, Denmark; 6 Department of Hematology, Aarhus University Hospital, Aarhus, Denmark; 7 Department of Hematology, Karolinska University Hospital, Stockholm, Sweden

**Keywords:** lymphoma, offspring, healthcare utilisation, survivorship, tree-based scan statistics

## Abstract

**Background and purpose:**

Advances in lymphoma treatment lead to a growing population of lymphoma survivors in childbearing ages who might be concerned about the impact of their disease on their children’s health. In this study, we aim to explore healthcare utilisation patterns that were associated with parental history of lymphoma.

**Patients/material and methods:**

Children born to lymphoma survivors (diagnosed over the period 2000–2018) were identified by linking the Swedish Cancer Register to national population registers. Each child born to a lymphoma survivor was matched on maternal age at childbirth to five children born to lymphoma-free parents. Information on in- and outpatient diagnoses and drug dispensations up to age five were obtained for all children.

**Results:**

We identified a total of 1,424 children born to lymphoma survivors and 7,120 matched children born to lymphoma-free parents. Children born to lymphoma survivors had a 8% higher healthcare utilisation rate (rate ratio: 1.08, 95% confidence intervals: 1.06–1.10) than other children. The panorama of diseases requiring healthcare utilisation was diverse and only one disease (International Classification of Diseases-10: H66, otitis media, unspecified) and one drug cluster (Anatomical Therapeutic Chemical: J07BC20, combination vaccine against hepatitis A and hepatitis B) was associated with a systematic difference (*p* < 0.05) when applying tree-based scan statistics.

**Interpretation:**

Children born to lymphoma survivors had slightly increased healthcare utilisation during early childhood. However, no strong or consistent disease- or drug-specific clusters explained this increase. Findings therefore suggest that the elevated healthcare use may reflect heightened health-seeking behaviour among cancer survivors, rather than underlying morbidity in their children. These results provide reassurance for lymphoma survivors considering parenthood.

## Introduction

Lymphomas or lymphoid neoplasms constitute a mixed group of tumours of B-/T-/NK-cell origin that are commonly classified based on their clinical presentation as indolent or aggressive, but more granular subtype classifications are often used as lymphomas are very heterogeneous in terms of biology, clinical presentation, treatment protocols and prognosis [1]. Approximately 2,100 new cases of lymphomas are diagnosed each year in Sweden [2]. In high-income countries, lymphomas represent the seventh or eighth most common malignancy across all ages, but also the fourth most common cancer among individuals 40 years or younger [3]. The relative high proportion of young lymphoma patients together with high overall survival lead to an increasing number of lymphoma survivors for whom family planning is a natural part of life [4].

Concerns about the health of future children are common among lymphoma patients [5]. Given that lymphomas originate in immune cells and that their treatment can have long-term systemic effects, transgenerational consequences for offspring health are biologically plausible. Most studies in this area so far have focused on birth outcomes, particularly low birth weight, in children born to lymphoma survivors motivated by its potential role in the aetiology of diseases later in life [6]. Yet, earlier studies in this field have reported conflicting results. While a previous systematic review concluded that young lymphoma and leukaemia survivors have an increased risk of pre-term delivery and delivering offspring with low birth weight [7], a recent large cohort study from the US did not confirm such differences [8]. There are no indications of increased risk of severe adverse perinatal outcomes, including stillbirth and birth defects [7, 8].

Although offsprings of lymphoma survivors are likely not at an increased risk of adverse perinatal outcomes, data on their subsequent health outcomes are lacking. A study on hospitalisation rates in children born to female cancer survivors (including lymphoma) found higher hospitalisation rates for children of female non-Hodgkin but not Hodgkin lymphoma survivors, compared to children born to females without cancer [9]. To the best of our knowledge, no study has reported if there are well-characterised disease groups that prompt an excess healthcare utilisation in offsprings of lymphoma survivors. Infections are one example of disease outcomes that could plausibly be increased in offsprings, owing to the immune-related pathology and treatment of lymphoma [9].

In this study, we aimed to explore healthcare utilisation patterns during the first 5 years of life comparing children with a mother or father who had a prior history of lymphoma to children born to lymphoma-free parents.

## Study population and methods

### Study population

This was a matched cohort study using Swedish population-based register data (Supplementary Figure S1). The study population of children born to lymphoma survivors and lymphoma-free individuals was obtained using a two-step procedure, in which we first identified the population of lymphoma survivors and comparators and, secondly, matched their children using a weighted matching procedure.

In the first step, we identified all males aged 18–55 years and females aged 18–45 years, diagnosed with lymphoma in Sweden between 2000 and 2018, and recorded in the Swedish Cancer Register (International Classification of Diseases [ICD]-10: C81–85, C88, C91.4). We then obtained 10 lymphoma-free general population comparators, concurrently matched on birthyear and sex, from *LymphomaBaSe*; a research platform constructed through linkage of several Swedish national health and population-based registers [10]. This linkage includes clinical and socioeconomical data on all lymphoma patients diagnosed in Sweden between 2000 and 2023, their matched lymphoma-free comparators from the general population, as well as their first-degree relatives.

In the second step, children with at least one parent diagnosed with lymphoma prior to their birth and children born to lymphoma-free comparators between 2000 and 2018 were identified using the Swedish Multi Generation Register. Due to the original matching procedure for constructing *LymphomaBaSe* (whereby comparators had been matched to patients with lymphoma), the demographic distribution of comparators and their children is not representative of the Swedish population during the study period. To restore the representativeness, we broke the original matching between comparators and lymphoma patients using inverse probability of sampling weights. To this end, we calculated Kaplan–Meier type weights stratified on the original matching factors for the comparators included in our study using annual population counts from Statistics Sweden [11, 12]. These were subsequently used in the weighted matching of children born to lymphoma survivors and children born to lymphoma-free parents on maternal age in order to restore the representativeness of children born in Sweden between 2000 and 2018.

Lastly, the analysis of drug dispensation clusters was conducted using a sub-cohort including only children born after 2005, based on the initiation of the Swedish prescribed drug register in July 2005.

### Outcome definitions

Our outcome of interest was healthcare utilisation, including both in- and outpatient visits and drug dispensations, from the day of birth and through the first 5 years of childhood. We focused on the first 5 years of life as all children in Sweden are regularly followed via Children’s Healthcare Centres up to age five. Information on date of visit and diagnoses (main and secondary diagnoses) represented in ICD-10 codes on all children’s in- and outpatient healthcare visits were obtained from the Swedish National Patient Register. Information on drug dispensations including date of dispensation and the associated Anatomical Therapeutic Chemical (ATC) code was obtained from the Swedish Prescribed Drug Register, not including drugs sold over the counter. For all analyses of in- and outpatient healthcare visits, we excluded the following ICD-10 chapters as events related to those chapters were unlikely to be connected to having a parent with lymphoma: Chapter XIX (S00–T98, injury, poisoning, and certain other consequences of external causes), Chapter XX (V01–Y98, external causes of morbidity and mortality), Chapter XXI (Z00–Z99, factors influencing health status and contact with health services). Moreover, due to data minimisation regulations applied by Statistics Sweden, we received information only on the block level for the ICD-10 chapters XVIII (Q00–Q99, congenital malformations, deformations and chromosomal abnormalities) and VIII (H60–H95, diseases of the ear and the mastoid system), and only on the chapter level for the ICD-10 chapters VII (H00–H59, diseases of the eye and adnexa) and XVI (P00–P96, certain conditions originating in the perinatal period). For the same reason, we were unable to fully characterise drug dispensations belonging to the following ATC classes due to data minimisation regulations applied by Statistics Sweden: D (alimentary tract and metabolism), P (antiparasitic products, insecticides and repellents), R (respiratory system), S (sensory organs), and V (various). Furthermore, we were limited to five-digit codes for the ATC classes A, B, C, G, H, L, M, N. However, codes from the ATC class J (anti-infectives for systematic use) were included with full detail.

### Time-to-event analysis

We estimated unadjusted rates of hospital visits (inpatient admissions and outpatient visits) and drug dispensation, comparing children born to parents with lymphoma and children born to lymphoma-free parents. Rate ratio (RRs) were estimated including all health care visits and drug dispensations during follow-up, with censoring at the date of death, emigration, or at 5 years, whichever came first. Incidence rate ratios (IRRs) reflecting time-to-first event were calculated additionally with censoring also at the first hospital visit/admission or first drug dispensation. The 95% confidence intervals (CI) and *p*-values were calculated using an exact method [13].

Mean number of hospital visits/admissions and drug dispensations in the presence of death as competing risk were estimated non-parametrically [14, 15].

### Tree-based scan statistic

Due to the explorative nature of this study, we further used tree-based scan statistics (TBSS), a method that has previously been proposed for signal detection in pharmacovigilance studies of drug safety [16, 17]. Unconditional Bernoulli TBSS were used to identify systematic patterns in healthcare utilisation, that manifest in terms of clusters of diagnosis codes across the ICD-10 and ATC classification code trees and where children born to parents with lymphoma were overrepresented when compared to children with lymphoma-free parents [16, 18]. In terms of estimation, the TBSS estimates log likelihood ratios that compare the observed to expected counts of first healthcare visits and drug dispensations in children born to lymphoma survivors for each possible cut along the Swedish ICD-10 and ATC tree, respectively [19]. The cuts were defined separately for the ICD-10 and ATC tree, respectively. Each chapter, block, category, and subcategory, included on the tree constituted a potential cut. *P*-values for each cut, adjusted for multiple testing, were calculated based on 9,999 Monte-Carlo simulations [18]. We ranked all cuts by their log likelihood ratios and reported the 10 most likely cuts with accompanying *p*-values, RRs, and rate differences. A more detailed description of TBSS can be found in the pre-registered statistical analysis plan associated with this study.

The analyses were carried out using the TreeMineR package available on CRAN, which we developed for this project (https://CRAN.R-project.org/package=TreeMineR). A statistical analysis plan was published on the Open Science Framework (https://osf.io/sbevn) prior to commencing the data analysis.

## Results

We identified 1,424 children born to lymphoma survivors between 2000 and 2018 of whom 651 (45.7%) and 773 (54.3%) were born to female and male lymphoma survivors, respectively (Table 1). The distribution of mothers’ and fathers’ highest education was relatively similar among children born to lymphoma survivors and those born to lymphoma-free parents. However, we found that the father’s age was above 41 years in 22% of children born to lymphoma survivors, whereas the corresponding proportion was 17% in children born to lymphoma-free parents. Child characteristics such as birth weight and birth order were similar in children born to lymphoma survivors and children born to lymphoma-free parents.

**Table 1:** Parents’ demographics of children born to lymphoma survivors between 2000 and 2018 in Sweden and children born to lymphoma-free comparators from the general population.

Characteristics of the study population	Children born to lymphoma survivors		Children born to lymphoma-free parents*	
	*N*	%^†^	*N*	%^†^
**Total**	1,424	100.0	7,120	100.0
**Females**	704	49.4	3,510	49.3
**Mother’s age at time of birth**				
18–30	461	32.4	2,308	32.4
31–40	889	62.4	4,431	62.2
41–50	74	5.2	381	5.4
**Father’s age at time of birth**				
18–30	276	19.4	1,554	21.9
31–40	836	58.7	4,336	60.9
41–50	273	19.2	1,047	14.7
51–60	32	2.3	130	1.8
60+	1	0.1	18	0.3
Unknown	6	0.4	35	0.5
**Mother’s years of education**				
< 10	78	5.5	450	6.3
10–12	439	30.9	2,188	30.7
> 12	904	63.5	4,448	62.5
Unknown	3	0.2	34	0.5
**Father’s years of education**				
< 10	150	10.5	593	8.3
10–12	545	38.3	3,048	42.8
> 12	720	50.6	3,420	48.0
Unknown	9	0.6	59	0.8
**Parent with lymphoma**				
Mother	651	45.8	-	-
Father	774	54.4	-	-
**Parent’s lymphoma type**				
HL	803	56.3	11	0.2
NHL	622	43.7	16	0.2
**Birth weight**				
≥ 2,500 g	1,348	94.7	6,826	95.9
< 2,500 g	73	5.1	289	4.1
Unknown	3	0.2	5	0.1
**Birth order**				
1	575	40.4	2,786	39.1
2	571	40.1	2,725	38.3
≥ 3	278	19.5	1,609	22.6

† Column-percentages.

* Including parents who developed lymphoma after their child were born.

**Figure 1:**
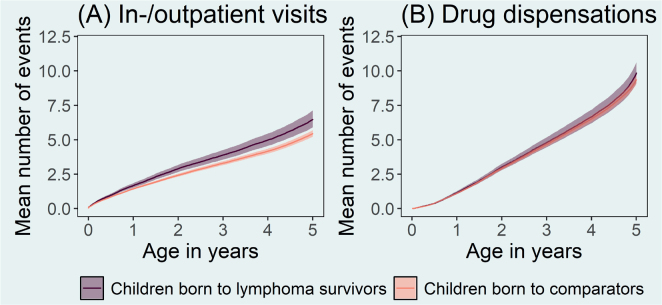
Mean number of (A) inpatient admissions and outpatient visits, and (B) drug dispensations up to age five in children born to lymphoma survivors between 2000 and 2018 in Sweden and children born to lymphoma-free comparators from the general population.

During the first 5 years of life, children born to lymphoma survivors had an increased overall healthcare utilisation, that is, including in- and outpatients visits and drug dispensations, compared to children born to lymphoma-free parents (RR: 1.08, 95% CI: 1.06–1.10). The rates of first inpatient admission, outpatient visit, and drug dispensation were 18% (RR: 1.18, 95% CI: 1.08–1.28), 20% (RR: 1.20, 95% CI: 1.17–1.23), and 2% (RR: 1.02, 95% CI 1.00–1.04) higher in children born to lymphoma survivors compared to children born to lymphoma-free parents, respectively (Table 2). This increase in the rate of inpatient admissions and outpatient visits among children born to lymphoma survivors translated into a higher number of average visits/admissions up to age five (Figure 1). When accounting for potential effect modification by parental sex we found that children whose father was diagnosed with lymphoma had increased rates of inpatient visits and hospital admission (RR: 1.11 95% CI: 1.06–1.17) as well as drug dispensations (RR: 1.10 95% CI: 1.06–1.14) compared to children whose mother was diagnosed with lymphoma (Supplementary Table S1). We did not find any differences in health care utilisation rates when comparing children born to a parent with a history of Hodgkin lymphoma versus non-Hodgkin lymphoma (Supplementary Table S2).

**Table 2:** Absolute counts and rates of inpatient admissions, outpatient visits and hospital visits combined (inpatient admissions and outpatient visits) as well as drug dispensations during the first 5 years of life among children born to lymphoma survivors and lymphoma-free parents.

Event	Health care utilisation within the first 5 years after birth				Rate ratio (95% CI)
	Children born to lymphoma survivors		Children born to comparators		
	Number	Rate^†^	Number	Rate^†^
**Incident events**					
Hospital visits combined	1,062	0.39	5,230	0.35	1.10 (1.03–1.18)
Outpatient visits	1,025	0.34	5,000	0.31	1.12 (1.05–1.20)
Inpatient admissions	385	0.07	1,751	0.06	1.14 (1.01–1.27)
Drug dispensations	1,182	0.54	5,791	0.50	1.08 (1.01–1.15)
**All events**					
Hospital visits combined	6,341	0.90	26,505	0.75	1.20 (1.17–1.23)
Outpatient visits	5,806	0.82	24,244	0.68	1.20 (1.17–1.23)
Inpatient admissions	673	0.09	2,869	0.08	1.18 (1.08–1.28)
Drug dispensations	9,843	1.46	47,753	1.44	1.02 (1.00–1.04)

† Incident and all event rates are estimated as the number of first and all events per person-years during the first 5 years after birth, respectively.

**Figure 2:**
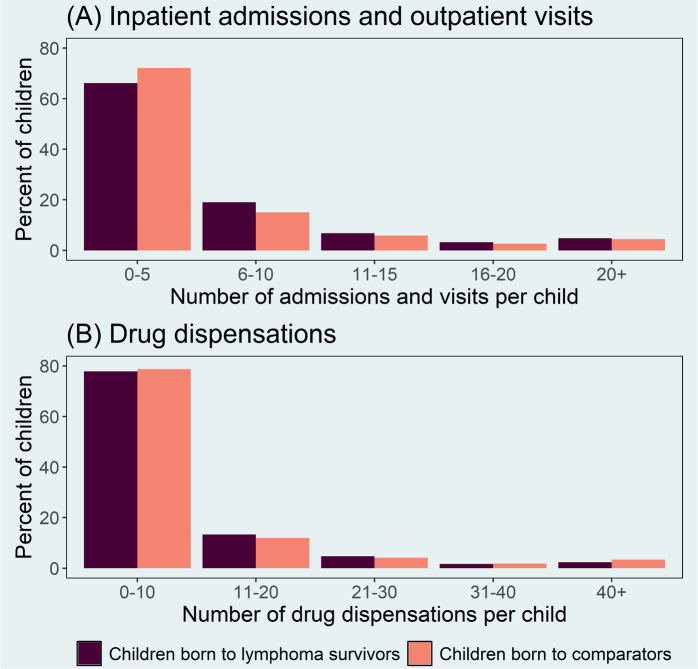
Number of (A) inpatient admissions, and (B) outpatient visits, and drug dispensations up to age five in children born to lymphoma survivors between 2000 and 2018 in Sweden and children born to lymphoma-free comparators from the general population.

The median number of hospital admissions and outpatient visits up to age five was 4 (inter quartile range [IQR]: 1–8) and 3 (IQR: 1–7) in children born to lymphoma survivors and children born to lymphoma-free parents, respectively (Figure 2). Corresponding median numbers of drug dispensations were 4 (IQR: 1–11) and 4 (IQR: 1–10). The proportion of children without any recorded inpatient admissions and outpatient visits was similar in children born to lymphoma survivors (15.2%) and children born to lymphoma-free parents (15.4%). For drug dispensations the corresponding proportions were 12.4 and 13.1%, respectively.

In the TBSS analysis, we identified only one disease and one drug cluster that was associated with differential healthcare utilisation signals (Table 3). Children born to lymphoma survivors had a 38% higher risk of being diagnosed with the ICD-10 code H66 (suppurative and unspecified otitis media) compared to children born to lymphoma-free parents (*p* = 0.049). The 5-year risk of suppurative and unspecified otitis media was 13.1 and 9.5% in children born to lymphoma survivors and children born to lymphoma-free parents, respectively. Additionally, we found that a higher proportion of children born to lymphoma survivors dispensed drugs of the ATC group J07BC20, which is a combination vaccine against hepatitis A and hepatitis B, compared to children born to lymphoma-free parents (*p* = 0.045). While 0.53% (*n* = 7) of children born to lymphoma survivors dispensed a combination vaccine against hepatitis A and hepatitis B up to age five, it was only 0.06% (*n* = 4) of children born to lymphoma-free parents.

**Table 3:** The 10 most likely clusters associated with health-care utilisation on the ICD-10 and ATC tree obtained from the tree-based scan statistic when ranked by their log-likelihood ratio.

Clusters of health-care utilisation		Children born to lymphoma survivors		Children born to comparators		Risk Ratio	Risk Difference	*P*-value^†^
		Events	5-year risk (%)	Events	5-year risk (%)			
**Cuts (ICD-10 codes)**								
H66	*Otitis media, unspecified*	186	13.06	675	9.48	1.38	3.58	0.049
H65–H75	*Diseases of middle ear and mastoid*	237	16.64	911	12.79	1.30	3.85	0.156
Ch. VIII	*Diseases of the ear and mastoid process*	261	18.33	1,025	14.40	1.27	3.93	0.217
Ch. I	*Certain infectious and parasitic diseases*	424	29.78	1,788	25.11	1.19	4.66	0.565
B25–B34	*Other viral diseases*	270	18.96	1,100	15.45	1.23	3.51	0.689
G00–G09	*Inflammatory diseases of the central nervous system*	8	0.56	9	0.13	4.44	0.44	0.711
D55–D59	*Haemolytic anaemias*	5	0.35	3	0.04	8.33	0.31	0.727
R620	*Delayed developmental milestone*	7	0.49	7	0.10	5.00	0.39	0.786
B34	*Viral infection of unspecified site*	266	18.68	1,091	15.32	1.22	3.36	0.824
M40–M54	*Dorsopathies*	17	1.19	35	0.49	2.43	0.70	0.829
**Cuts (ATC codes)**								
J07BC20	*Hepatitis A/B vaccination*	7	0.53	4	0.06	8.64	0.47	0.045
J07B	*Viral vaccines*	34	2.59	88	1.36	1.91	1.23	0.223
B03BB	*Folic acid and derivatives*	5	0.38	3	0.05	8.23	0.33	0.304
J07	*Vaccines*	50	3.81	156	2.41	1.58	1.40	0.456
J01DD14	*Ceftibuten*	30	2.29	85	1.31	1.74	0.98	0.779
J01DD	*Third-generation cephalosporins*	31	2.36	89	1.37	1.72	0.99	0.783
J01EA	*Trimethoprim and derivatives*	58	4.42	195	3.01	1.47	1.41	0.784
J01EA01	*Trimethoprim*	58	4.42	195	3.01	1.47	1.41	0.784
B03B	*Vitamin B12 and folic acid*	5	0.38	5	0.08	4.94	0.30	0.850
J07BC	*Hepatitis vaccines*	10	0.76	18	0.28	2.74	0.48	0.867

† *P*-values for cluster significance obtained from Monte-Carlo simulations and adjusted for multiple-testing.

ICD: International Classification of Diseases; ATC: Anatomical Therapeutic Chemical; Ch: ICD-10 chapter.

In terms of statistical power, the TBSS analyses were able to detect disease and drug clusters with risk ratios of at least 1.37, and 1.34, respectively, for clusters with a high prevalence (10%). Clusters with lower prevalence (0.5%) were detectable if the corresponding risk ratios were greater than 2.99 and 2.80 (Supplementary Figure S2).

## Discussion

The aim of this study was to explore differences in healthcare utilisation patterns among children born to lymphoma survivors compared to children born to lymphoma-free parents. We hypothesised that both the lymphoma disease itself as well as lymphoma treatment might have transgenerational effect which might impact offspring health. Although we observed slightly higher rates of inpatient admissions, outpatient visits, and drug utilisation overall in the former group, this excess healthcare utilisation was distributed across a panorama of different diseases and drug groups. Using TBSS, no strong signals for specific disease clusters or dispensed drugs were detected. The only (borderline) significant associations between having a parent with a medical history of lymphoma and higher use of healthcare during the first 5 years of life were indicated by more frequent occurrence of ear infections (inpatient admissions and outpatient visits) and hepatitis vaccinations (drug dispensations), both unlikely to be of clinical relevance.

Our findings of higher overall rates of inpatient admissions and outpatient visits are in line with a previous study using Swedish register data. In their study the authors reported an overall increased hospitalisation rate throughout life in children born to survivors of non-Hodgkin lymphoma, but no corresponding elevated rate among children born to Hodgkin lymphoma survivors [9]. When studying hospitalisation rates of children born to cancer patients irrespectively of cancer type, the authors could not identify a specific disease group that prompted this elevated rate among a set of pre-specified disease groups, except for neoplasms [9]. This previous investigation was, however, not limited to early childhood and did not study or report healthcare utilisation with the same granularity in terms of ICD and ATC codes that the data-driven approach applied in the present study was able to do.

A possible explanation of why we could not identify any disease and drug clusters prompting an increase in healthcare utilisation despite observing a general excess healthcare utilisation in children born to lymphoma survivors could be due to differences in healthcare seeking behaviour of cancer survivors compared to individuals without a history of cancer. Cancer survivors naturally require more frequent contacts with the healthcare system for several reasons, including late effects of their treatments and disease, concerns or symptoms associated with cancer recurrence, and the close monitoring during the period of their cancer treatment [20, 21]. Previous research showed that Hodgkin lymphoma survivors diagnosed in Sweden have an excess healthcare utilisation years after completing their treatment compared to individuals without a history of cancer [22]. The close connection of cancer survivors to healthcare services, combined with an overall high concern of negative effects of their disease and treatment on their offspring’s health, might also prompt extra healthcare visits regarding their offspring’s health. In case the health seeking behaviour is independent of their children’s symptoms, such additional visits would manifest as higher rates of healthcare use but not limited to specific disease or drug clusters. The increased dispensation of vaccinations against hepatitis A and B can also be seen as a negative outcome in support of this hypothesis.

A main strength of our study is its agnostic and hypothesis generating analysis framework that enabled us to explore 11,172 potential disease and 2,406 drug clusters associated with healthcare utilisation patterns. Also, all reported *p*-values were estimated taking the full tree and all potential comparisons within the tree into account via a simulation-based approach that incorporated adjustment for multiple testing by design [18]. However, the adjustment for multiple testing comes at the cost of statistical power. Due to the high number of comparisons made, we were only able to detect disease and drug clusters with a moderate to high excess risk in children born to lymphoma survivors compared to children with lymphoma-free parents. A more detailed analysis of children born to parents with specific lymphoma subtypes was, hence, not possible.

Additionally, our study faces limitations concerning the registers used in the analysis. The in- and outpatient registers do not capture primary care visits. Hence, we were unable to investigate health enquiries that did not require specialist in- or outpatient care. As for less severe health conditions we were thus limited to information recorded in the prescribed drug register (available after 2005).

In conclusion, we found that children of lymphoma survivors had higher healthcare utilisation up to age five compared with those of lymphoma-free parents, but no strong associations with specific disease or drug groups were observed. The moderate sample size, however, limited the possibility to detect small disease-specific effects. Overall, these findings suggest that parental lymphoma and its treatment do not markedly impair offspring health, which should be reassuring for young survivors.

## Statements & declarations

### Competing interests

I Glimelius received funding from the department for participating in educational sessions arranged by Johnson and Johnson and Abbvie, and participated in a real-world data collaboration project funded by Takeda. All COI are unrelated to the current study.

### Author contributions

**Joshua P. Entrop:** Conceptualisation, Methodology, Software, Formal Analysis, Data Curation, Writing – Original Draft. **Viktor Wintzell:** Conceptualisation, Methodology, Software, Writing – Review & Editing. **Caroline E. Dietrich:** Conceptualisation, Writing – Review & Editing. **Ingrid Glimelius:** Conceptualisation, Writing – Review & Editing. **Tarec C. El-Galaly:** Conceptualisation, Writing – Review & Editing. **Karin E. Smedby:** Conceptualisation, Writing – Review & Editing, Resources. **Sandra Eloranta:** Conceptualisation, Methodology, Writing – Original Draft, Supervision, Funding Acquisition.

### Ethical approval

This study has been approved by the Regional Ethical Review Board in Stockholm (No. 2019-00242 and 2020-04872).

### Data availability

The data that support the findings of this study are not openly available due to reasons of sensitivity and are available from the corresponding author upon reasonable request. Data are located in controlled access data storage at Karolinska Institutet.

## Supplementary Material


