# Supplementary material for: Utilisation of healthcare in children born to lymphoma survivors in Sweden
Source: Acta Oncol. 2025 Nov 23;64:43950. doi: 10.2340/1651-226X.2025.43950 (PMC12659140; doi:10.2340/1651-226X.2025.43950)
Supplement: Supplementary file 1 [file AO-64-43950-s1.pdf]

Supplementary material has been published as submitted. It has not been copyedited, or typeset by Acta Oncologica

Supplement

Utilization of Healthcare in Children Born to Lymphoma Survivors in Sweden

Joshua P. Entrop, Viktor Wintzell, Caroline E. Dietrich, Ingrid Glimelius, Tarec C. El-Galaly, Karin E. Smedby, and Sandra Eloranta

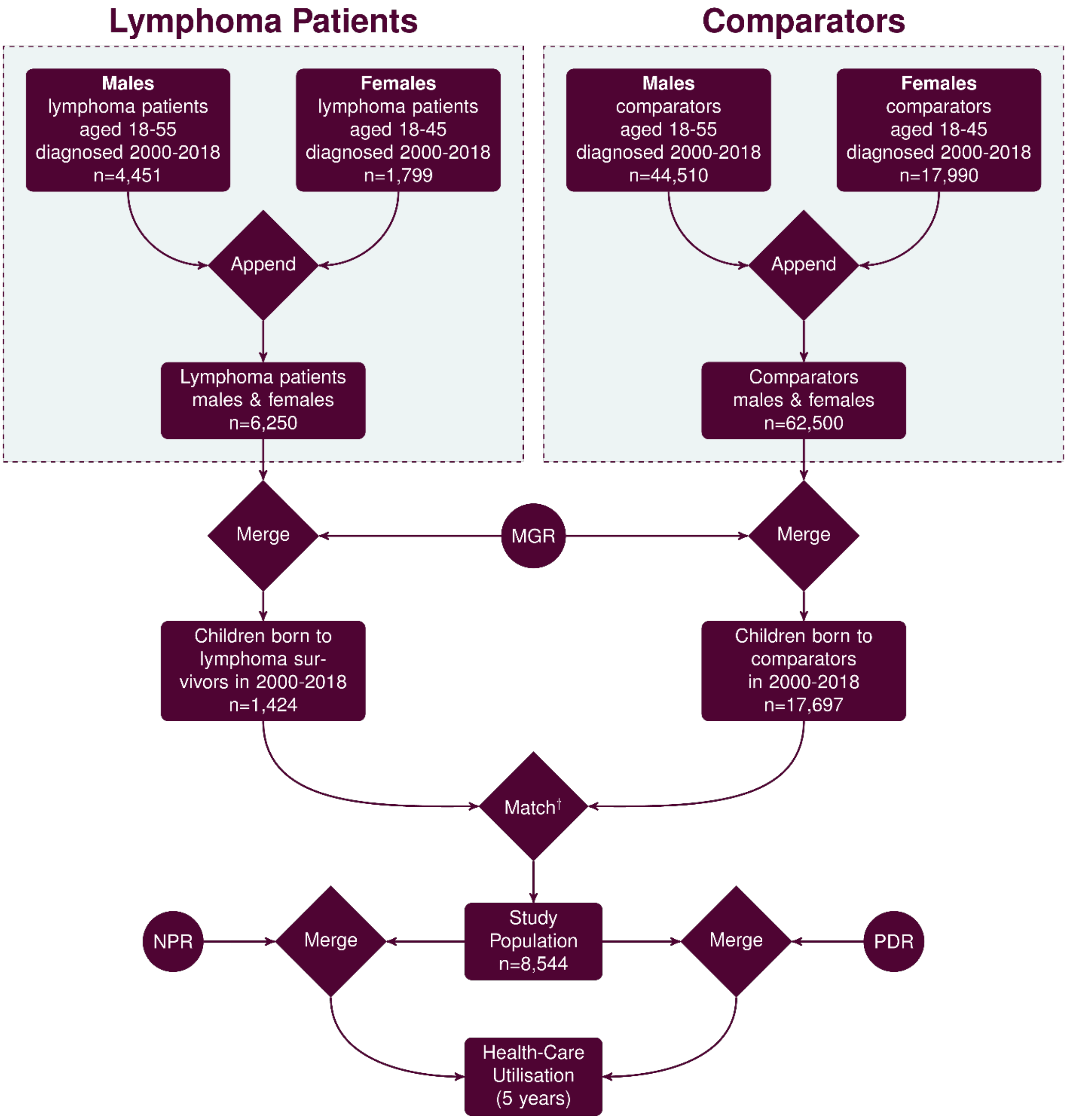

Figure S1 Flowchart of the study population of children born to female and male lymphoma survivors and comparators in Sweden between the years 2000-2018.
†: weighted frequency matching on mothers' birth year.
Abbreviations: MGR: multi generation register. NPR: national patient register. PDR: prescribed drug register

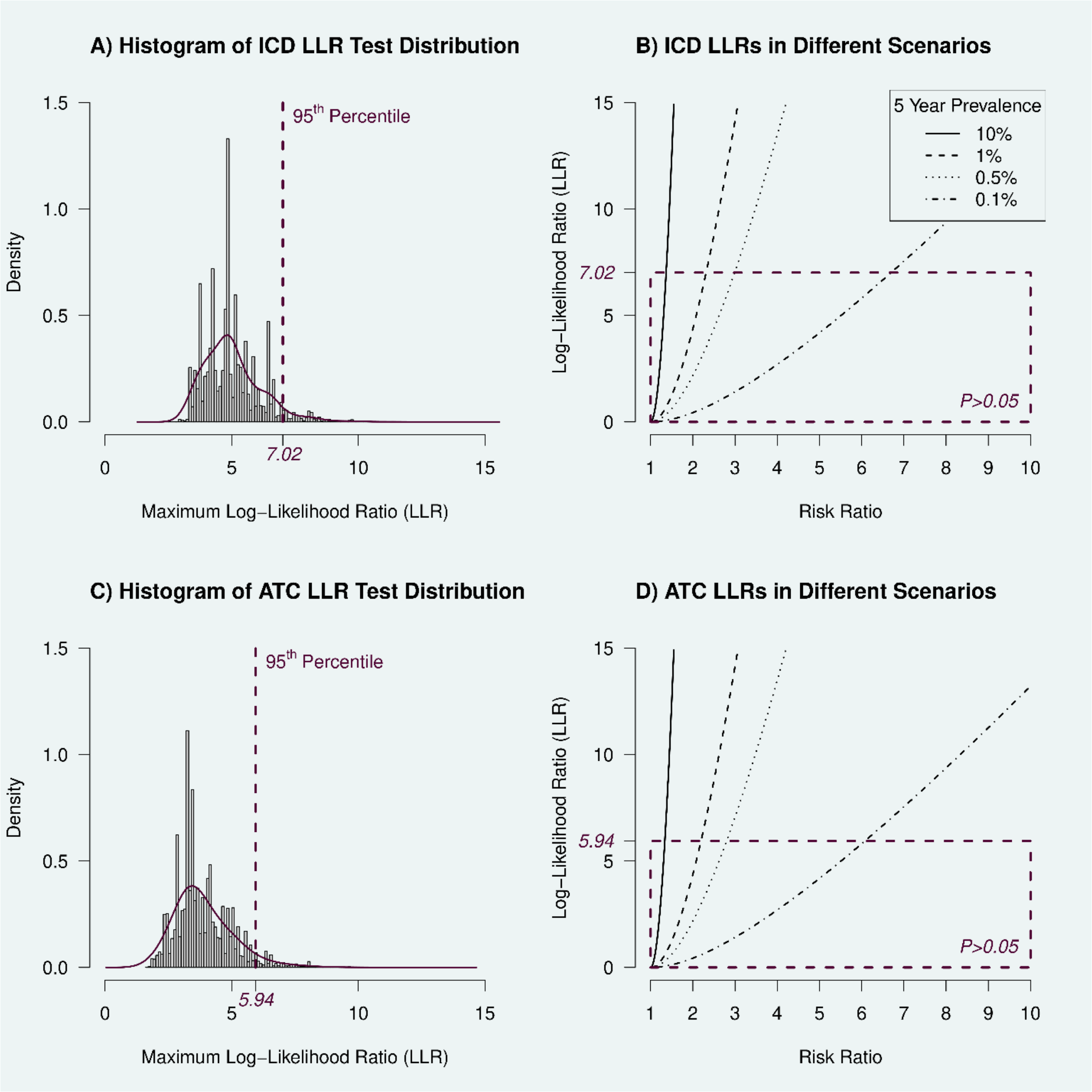

Figure S2 Panels (A) and (C) show the log-likelihood ratio (LLR) test distribution of the maximum LLRs in each Monte-Carlo simulation across the ICD and the ATC tree, respectively. The dashed line shows the P<0.05 cut-off. Panels (B) and (D) show LLRs across different scenarios assuming a study population of 1 424 exposed and 7 120 unexposed individuals.

Interpretation Supplement Figure S2 shows the least detectable risk ratios in the TBSS analysis across different scenarios of disease/drug dispensation prevalence based on the presented data. For example, panel B shows that given a prevalence of 0.01% a risk ratio of 6 would not be detectable, as the corresponding log-likelihood ratio is below the threshold of 7.02 indicated by the dashed box, whereas a risk ratio of 8 would be detectable as the corresponding log-likelihood ratio is greater than the threshold of 7.02 indicated by being outside the dashed box. The analysis showed that we were able to detect disease and drug clusters with risk ratios of at least 1.37, and 1.34, respectively, for clusters with a high prevalence (10%). Clusters with lower prevalence (0.5%) were detectable if the risk ratio was great than 2.99 and 2.80 for disease and drug clusters, respectively.

	Health care utilisation within the first 5 years after birth in children born to lymphoma survivors				
	Children born to fathers with lymphoma		Children born mothers with lymphoma		Rate ratio (95% CI)
	Number	Rate	Number	Rate	
**All events**					
Hospital visits combined	3,612	0.94	2,729	0.84	1.11 (1.06-1.17)
Drug dispensations	5,575	1.41	4,268	1.32	1.10 (1.06-1.14)

Table S1 Absolute counts and rates of hospital visits combined (inpatient admissions and outpatient visits) as well as drug dispensations during the first 5 years of life among children born to lymphoma survivors by type of parent diagnosed with lymphoma.

	Health care utilisation within the first 5 years after birth in children born to lymphoma survivors				
	Children born to a parent with Hodgkin lymphoma		Children born to a parent with non-Hodgkin lymphoma		Rate ratio (95% CI)
	Number	Rate	Number	Rate	
**All events**					
Hospital visits combined	3,596	0.90	2.745	0.89	1.01 (0.96-1.07)
Drug dispensations	5,578	1.40	4,265	1.38	1.01 (0.97-1.05)

Table S2 Absolute counts and rates hospital visits combined (inpatient admissions and outpatient visits) as well as drug dispensations during the first 5 years of life among children born to lymphoma survivors by lymphoma type of the parent diagnosed with lymphoma.
